# MiR-29c-3p, a target miRNA of LINC01296, accelerates tumor malignancy: therapeutic potential of a LINC01296/miR-29c-3p axis in ovarian cancer

**DOI:** 10.1186/s13048-020-00631-w

**Published:** 2020-03-19

**Authors:** Hui Xu, Hong-Luan Mao, Xin-Rui Zhao, Yue Li, Pei-Shu Liu

**Affiliations:** 1 Department of Gynecology, QiLu Hospital of Shandong University, No. 107 Wenhua Xi Road, Jinan, Shandong 250012 People’s Republic of China; 2 Department of Gynecology, The Second Hospital of Shandong University, Jinan, 250033 People’s Republic of China

**Keywords:** LINC01296, miR-29c-3p, EMT, Ovarian cancer

## Abstract

As one of the main gynecological cancers, ovarian cancer (OC) has an unfavourable outcomes owing to its high recurrence and metastasis rate. Our previous studies have revealed that LINC01296 functions as an oncogene in OC, but the underlying mechanism has not been explored. The aim of this paper was to further investigate that how LINC01296 plays a role in OC. Through online software prediction, miR-29c-3p has been discriminated as the target miRNA of LINC01296 for further research, and subsequent luciferase assay confirmed bioinformatics prediction. Then the data obtained from the two databases (GSE119055 and GSE83693) were analyzed by GEO2R for differential gene analysis. The results indicated that the miR-29c-3p was lowly expressed in OC tissues than that in normal ovarian tissues, and its expression in recurrent OC tissues was lower than that in primary OC tissues. Simultaneously, Kaplan-Meier survival analysis illustrated that the lower expression of miR-29c-3p was interrelated to unfavourable outcomes of OC. Further, the qRT-PCR data revealed that the miR-29c-3p expression in OC cell lines (SKOV-3 and OVCAR-3) was markedly declined than that in normal control cells (IOSE80). Subsequently, the functional experiments, such as CCK8, colony formation and Transwell assays, prompted that inhibition of miR-29c-3p can obviously increase the proliferation, invasion and migration of OVCAR3 and SKOV3 cells compared with control group, while downregulation of LINC01296 showed an opposite result. It is worth noting that downregulation of LINC01296 can reverse the effect of miR-29c-3p suppression on OC cells. Finally, we detected the changes of EMT-related proteins by western blot experiment, and reached a similar conclusion that knockdown of LINC01296 reversed the EMT caused by miR-29c-3p inhibition. In sum up, the cancer-promoting function of LINC01296 was achieved by regulating the expression of miR-29c-3p, and LINC01296/miR-29c-3p axis mediates the mechanical regulation of EMT in OC cells, hoping to provide the novel biomarkers and possibilities for OC therapy.

## Introduction

Ovarian cancer (OC), as one of gynecological malignant tumors, has the characteristics of high incidence, high recurrence rate and high mortality [1, 2]. Due to the limitations of its diagnostic methods, it is difficult to diagnose the disease in the early stage, and 60% of patients are found in the advanced stage [3]. Up to now, chemotherapy is the main treatment for OC, but the drug resistance and strong metastasis of OC result in poor outcomes [4]. The five-year survival rate was 47% for all patients, while it was as low as 20% for patients with stage IV [2]. Lack of effective biomarkers for early OC diagnosis is the main reason for low survival rate [5], so it is urgent to figure out the pathological mechanism of OC and find effective molecular targets for diagnosis and treatment. In our previous studies, we found that long intergenic non-coding RNA 01296 (LINC01296) can be used as a biomarker for diagnosis and prognosis of OC patients [6], but its mechanism remains unclear.

Recently, the effect of LncRNAs on the microRNAs (miRNAs) function has become a research hotspot [7]. Extensive researches have found that LncRNAs regulated the progression of cancer by interacting with miRNAs, due to it can act as a sponge of miRNA to regulate its activity [8–10]. The regulatory mechanism of LINC01296 has been reported in various cancers. Xuefei Hu et al. found that LINC01296 accelerated the development of non-small cell lung cancer by inhibiting the expression of miR-5095 [11]. In colon carcinoma, miR-21a combines with LINC01296 to reverse its carcinogenic effect [12]. In addition, LINC01296/miR-122-5p axis regulate the development of hepatocellular carcinoma through EMT pathway [13]. These findings provided an insight for us to explore how LINC01296 implicated in the progression of OC.

Therefore, we used the target gene prediction website to predict the target miRNA of LINC01296, and confirmed miR-29c-3p as the potential target miRNA of LINC01296 for further study. Recently, as a key miRNA, miR-29 has been reported to be involved in various major process of cancers, such as proliferation, apoptosis and metastasis, *etc* [14]*.* MiR-29c-3p, a member of the miR-29 family, was a potential tumor suppressor and it was down-regulated in several types of human cancers [15]. However, its role in OC remains to be elucidated.

Based on the references and our previous study, we planned to in-depth understand the regulatory relationship between LINC01296 and miR-29c-3p, and whether LINC01296/ miR-29c-3p axis can contribute to the pathogenesis of OC, hoping to provide a potentially effective method for targeted diagnosis and treatment of OC.

## Materials and methods

### Bioinformatics prediction

The target miRNA of LINC01296 was predicted by target gene prediction and analysis website miRBase (http://www.mirbase.org/) and starBase (http://starbase.sysu.edu.cn/).

### Data collection

Gene expression profiles of OC patients were downloaded from the two independent datasets (the accessing number of GSE119055, and GSE83693) of Gene Expression Omnibus (GEO, http://www.ncbi.nlm.nih.gov/geo/). GSE119055 dataset contained 6 OC tissue samples and 3 normal ovarian tissue samples, and GSE83693 included 8 recurrent OC tissue samples, 8 primary OC tissue samples and 4 normal ovarian tissue samples.

### Cell lines and cell transfection

Human OC cell lines (SKOV-3 and OVCAR-3) were purchased from American Type Culture Collection (ATCC), and the normal control cell (IOSE80) were obtained from Shanghai Cell Bank, Chinese Academy of Medical Sciences. RPMI-1640 medium (Sigma, St. Louis, MO) were used to routinely culture the cells in a 37 °C incubator with 5% CO_2_, which containing 10% fetal bovine serum (FBS, Gibco, CA, USA), streptomycin (0.1 mg/ml) and penicillin (100 U/ml).

MiR-29c-3p mimics or inhibitors were transfected into cells to implement ectopic expression of miR-29c-3p, and LINC01296 siRNA was used to knockdown LINC01296. The miR-29c-3p inhibitors (50,100,150 nM), miR-29c-3p mimic and negative control, and si-LINC01296#1(5′-CUGAAACAUAUUCCGUGGUTT-3′), si-LINC01296#2(5′-GGCUGGAGAAUAUUUCCUATTTT-3′) and si-con (5′-AATTCTCCGAACGTGTCACGT-3′) were synthesized by Shanghai Genepharma Co., Ltd. (Shanghai, China). Lipofectamine2000 (Invitrogen, CA, USA) was used for transfection according to the standard of supplier. After 48 h, transfected cells were used for further experiments.

### RNA extraction and qRT-PCR

Total RNA was extracted from cell lines using Trizol reagent (Invitrogen, CA, USA), and then reverse transcription was performed to form cDNA. After that, so as to test the expressions level of LINC01296 and miR-29c-3p, qRT-PCR was performed using SYBR Green supermix and Hairpin-it TM miRNAs qPCR kit (Genepharma, Shanghai, China), based on the manufacturer’s instructions. GAPDH and U6 were used as internal control, and 2^-ΔΔCT^ method was applied to calculate the data.

The primer sequences were as below:

LINC01296: F: 5′- AAGTGGCACCAGCCTCACT -3′

R: 5′- CGGCCAAGT TCTTTACCATC -3′,

GAPDH: F: 5′- GGAGCGA GATCCCTCCAAAAT -3′

R: 5′- GGCTGTTGTCATACTTCTCATGG-3′

miR-29c-3p F: 5′-GCTGGTTTCATATGGTGG -3′

R: 5′-GAACATGTCTGCGTATCTC -3′

U6: F: 5′-CTCGCTTCGGCAGCACATATACT-3′

R:5′-ACGCTTCACGAATTTGCGTGTC-3′

### Western blotting assay

The cells were dissolved in RIPA buffer, and M-per mammal protein extraction reagent (Thermo Scientific, USA) was utilized to extract their proteins. The protein was dissociated on the SDS-PAGE microgel and then transferred to the PVDF membrane. Subsequently, the membrane was incubated with primary antibodies overnight at 4°Cfollowed by washing 3 times with TBST. Afterwards, the secondary antibodies were used to incubate the blots at room temperature for 1 h. Finally, ECL Substrates (Millipore, MA, USA) was used to observe the signals.

### Cell counting Kit-8 (CCK-8) assay

CCK-8 assay was conducted to measure the cell viability. Cells were inoculated into 96-well plates at a density of 1*10^4^ and then conventional cultured in carbon dioxide incubator for 24 h, 48 h and 72 h respectively. Notably, added 10 μl of CCK-8 reagent to each well at 2 h before the predefined time-points. Afterwards, a microplate reader was used to measure the OD value at 450 nm, which can assess the cell viability.

### Colony formation assay

Colony formation assay was performed to measure the colony formation ability of OC cells. About 400 cells were inoculated in culture dish containing 5 ml pre-heated medium. Cells were cultured for 1–2 weeks in a cell incubator at 37°Cwith 5% CO_2_ and saturated humidity until naked-eye-visible clones appeared. The cells were fixed with 4% paraformaldehyde 5 ml for 30 min. Then remove the fixative and dye the cells with 0.1% crystal violet dye for 30 min. Ultimately, numbers of colony were counted.

### Transwell assays

The migration and invasion experiments were conducted in a 24-well transwell chambers. The Matrigel was used to pre-coat the upper surface of chambers for invasion assay, and the migration assay do not require gluing treatment. For each assay, cells was added to the upper chamber at the density of 5*10^5^ with serum-free medium after 24 h transfection, simultaneously, 500 ml serum medium was putted to the lower chamber as the chemical attractant. After incubation overnight, cells in the upper surface of chambers were wiped off with cotton swabs, and the migrated or invaded cells on the lower surface of chambers were fixed with 4% paraformaldehyde for 30 min and then stained with 0.1% crystal violet for 15 min. After that, five visual fields were stochastic picked under the microscope and cell numbers were counted.

### Luciferase reporter assay

Luciferase reports assay were carried out to further confirm whether LINC01296 could directly interact with miR-29c-3p. The wild type and mutant type 3’UTR of LINC01296 were cloned into pmirGLO luciferase vector to construct luciferase expression plasmids WT-LINC01296 and MUT-LINC01296, respectively, and co-transfected them into HEK-293 T cells with miR-29c-3p mimic or miR-29c-3p mimic NC. Lipofectamine 2000 was used for transfection following the instructions. After 48 h, a luminometer (Beckman Coulter LD400, CA, USA) was used to detect the luciferase activity.

### Statistical analysis

The data was analyzed using SPSS22.0 statistical analysis software. T-test was used to compare the differences between the two groups, and One-way ANOVA analysis and post-test of Tukey were used to compare the differences among three groups and above. Kaplan-Meier survival analysis was used to plot the survival curves, and the high-expression group and the low-expression group were grouped according to the median expression level of miR-29c-3p. *P* < 0.05 was considered to be significantly different.

## Results

### MiR-29c-3p was predicted to be the target miRNA of LINC01296, which was lowly expressed in OC tissues and cell lines, causing unfavourable outcomes in OC

**Fig. 1:**
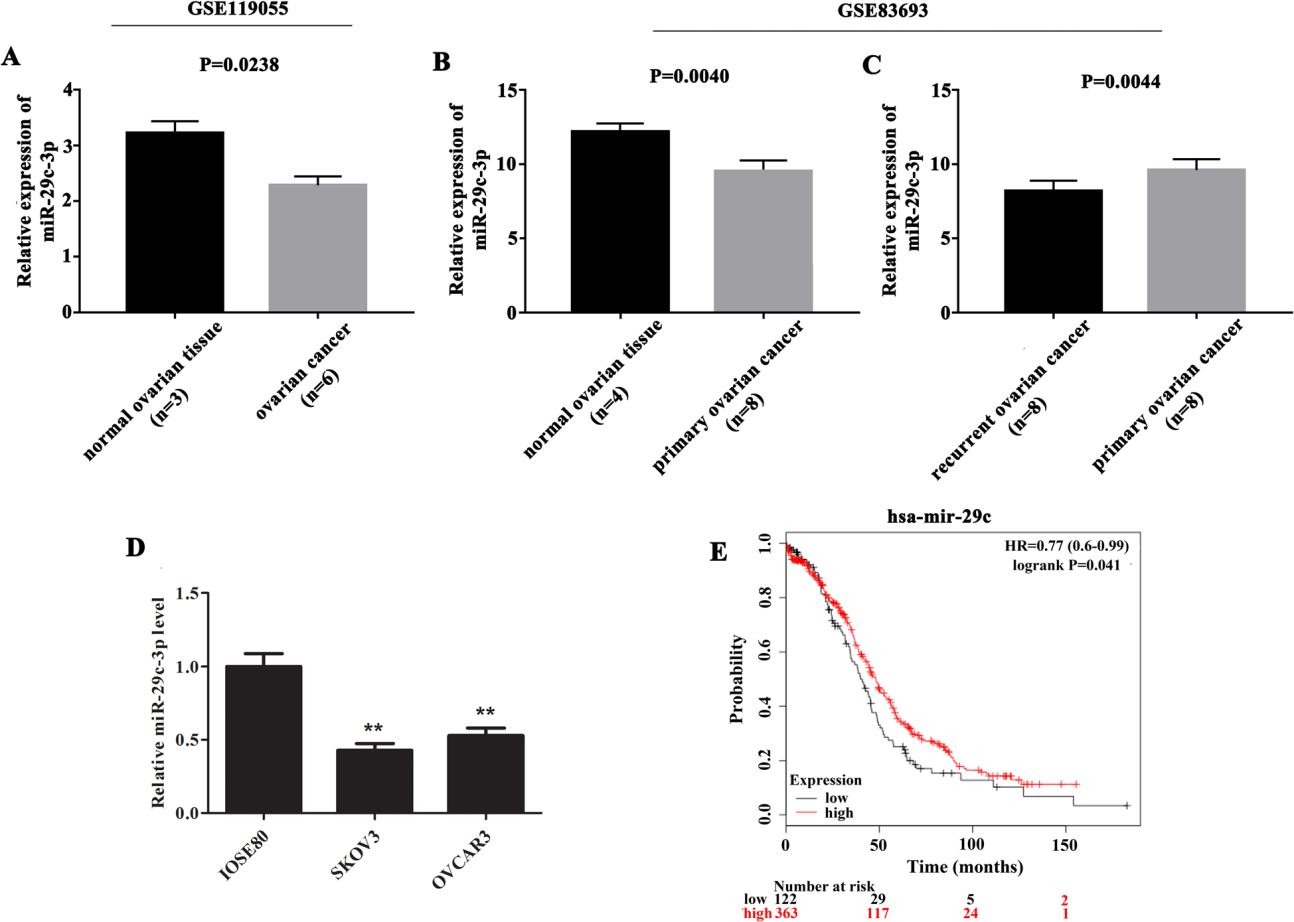
Expression of miR-29c-3p in OC. **a** MiR-29c-3p was lowly expressed in OC tissues compared with normal samples (*P* = 0.0238 for GSE119055). **b** The miR-29c-3p expression was clearly decreased in primary OC tissues than that in normal OC tissues (*P* = 0.0040 for GSE83693). **c** The expression of miR-29c-3p in recurrent OC tissue was markedly declined than that in primary OC tissues (*P* = .0044 for GSE83693). **d** qRT-PCR assay illustrated that the expression of miR-29c-3p in two OC cells (SKOV3 and OVCAR3) was down-regulated than that in corresponding normal cell (IOSE80). **P < 0.01. **e** Down-regulation of miR-29c-3p was interrelated to unfavourable outcomes in OC (*P* = 0.041)

Firstly, StarBase website (http://starbase.sysu.edu.cn/) was used to predict the target miRNA of LINC01296. Based on bioinformatics analysis, miR-29c-3p has been discriminated as the target miRNA of LINC01296 for further research. After that, differentially expressed genes were identified using an online tool, GEO2R. From the analysis of data in GSE119055 database, we found that the expression of miR-29c-3p was obviously reduced in OC tissue than that in the normal ovarian tissue (Fig. 1a, *P* < 0.05). Simultaneously, after analyzing the data collected from the GSE83693 database, we can see that miR-29c-3p was lowly expressed in primary OC tissue than that in normal ovarian tissue (Fig. 1b, *P* < 0.01), and its expression in recurrent OC tissue was markedly lower than that in primary OC tissue (Fig. 1c, *P* < 0.01). Afterwards, qRT-PCR assay was carried out to analyze the miR-29c-3p expression in OC cell lines. The results revealed that miR-29c-3p expression was obviously decreased in OC cell lines (SKOV3, OVCAR3) than that in the normal cells (IOSE80) (Fig. 1d, *P* < 0.05). These results exhibited that miR-29c-3p was down-regulated in OC, and it was remarkable that with the development of OC, the expression of miR-29c-3p was decreased.

Moreover, so as to study the prognostic value of miR-29c-3p in OC, Kaplan-Meier survival analysis was performed. The result in Fig. 1e showed that there was a conspicuous discrepancy in survival rate between low-expression group and high-expression group, and those patients with low expression of miR-29c-3p had lower overall survival (*P* < 0.05).

### LINC01296 directly targets miR-29c-3p and negatively regulated its expression

**Fig. 2:**
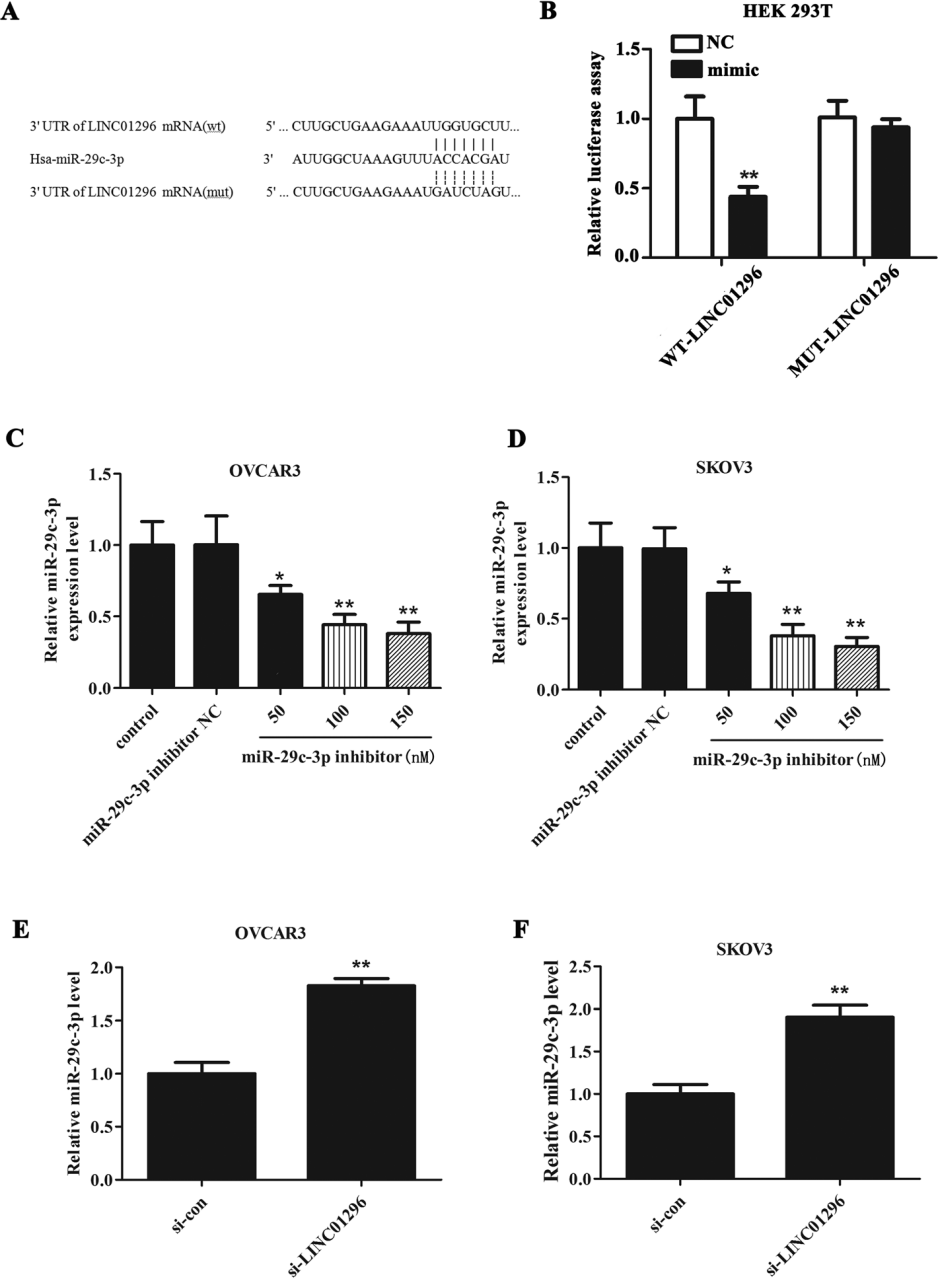
LINC01296 directly targets miR-29c-3p. **a** The binding sequence showed that 3′-UTR of LINC01296 contains the binding site of miR-29c-3p; **b** The fluorescence intensity of WT-LINC01296 and miR-29c-3p mimic co-transfected HEK-293 T cells was significantly declined (*P* < 0.01), while the fluorescence intensity of MUT-LINC01296 and miR-29c-3p mimic co-transfected HEK-293 T cells was almost unchanged. (**c**-**d**) After OVCAR3 and SKOV3 cells were treated with different concentration (50, 100 and 150 nM) of miR-29c-3p inhibitor, we can see that miR-29c-3p inhibitor had notable inhibiting effect on the miR-29c-3p expression when the concentration was 50 (*P < 0.05 vs. control), 100 (**P < 0.01 vs. control) and 150 nM (**P < 0.01 vs. control). (**c**-**d**) The expression of miR-29c-3p in SKOV3 and OVCAR3 cells transfected with si-LINC01296 was markedly elevated. **P < 0.01 vs. si-con group

In order to validate the prediction that miR-29c-3p was the target miRNA of LINC01296, we conducted a dual-luciferase reporter assay. The 3’UTR of LINC01296 containing predicted binding sites or mutations were shown in Fig. 2a. The 3’UTR of LINC01296 was cloned into the luciferase reporter gene to construct WT-LINC01296, and the mutant version (MUT- LINC01296) was also constructed via binding site mutation. Subsequently, HEK-293 T cells were co-transfected with WT-LINC01296 vector or MUT- LINC01296 and miR-29c-3p mimic or miR-29c-3p mimic NC, and the results showed that over-expression of miR-29c-3p markedly reduced the luciferase activity in the WT-LINC01296 vector, and the mutant presumed binding site can block the miR-29c-3p-mediated inhibition of luciferase activity (Fig. 2b). In addition, OVCAR3 and SKOV3 cells were treated with different concentration (50, 100 and 150 nM) of miR-29c-3p inhibitor to detect the inhibition efficiency. It can be seen from Fig. 2c-d that miR-29c-3p inhibitor had notable inhibiting effect on the miR-29c-3p expression when the concentration was 50 (*P* < 0.05), 100 (*P* < 0.01) and 150 nM (*P* < 0.01). Wherein, the inhibition efficiency at 100 nM was exceeded 50%, which has reached the standard of inhibitor use, and there was no significant difference in the inhibition effect between 150 nM and 100 nM. Hence, 100 nM was selected as the working concentration in the subsequent assays. Furthermore, from Fig. 2e-f we can see that knockdown of LINC01296 leaded to a promotion of miR-29c-3p expression in OVCAR3 and SKOV3 cells. Overall, these results indicated that LINC01296 can negatively regulate the miR-29c-3p expression at the transcriptional level.

### The influence of LINC01296/miR-29c-3p axis on the biological behaviour of OC cells

**Fig. 3:**
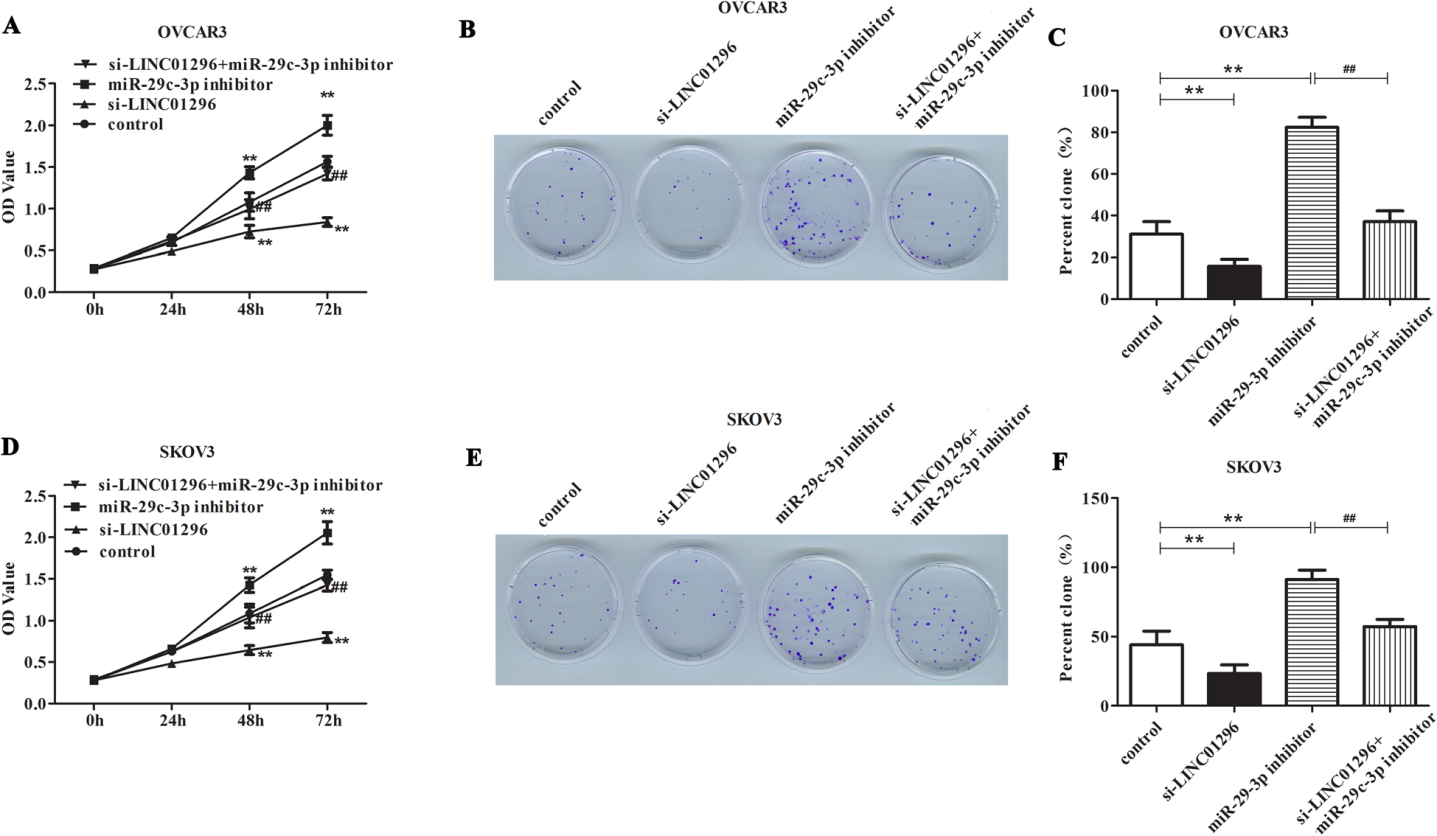
The influence of LINC01296/ miR-29c-3p axis on the proliferation of OC cells. The SKOV3 and OVCAR3 cells were treated with miR-29c-3p inhibitor, si-LINC01296, or miR-29c-3p inhibitor plus si-LINC01296. CCK8 assay (**a**, **d**) and Colony formation assay (**b**-**c**, **e**-**f**) were performed to detect the proliferation of OC cells in different conditions. **P < 0.01 vs. control group, ^##^*P* < 0.01 vs. miR-29c-3p inhibitor group

**Fig. 4:**
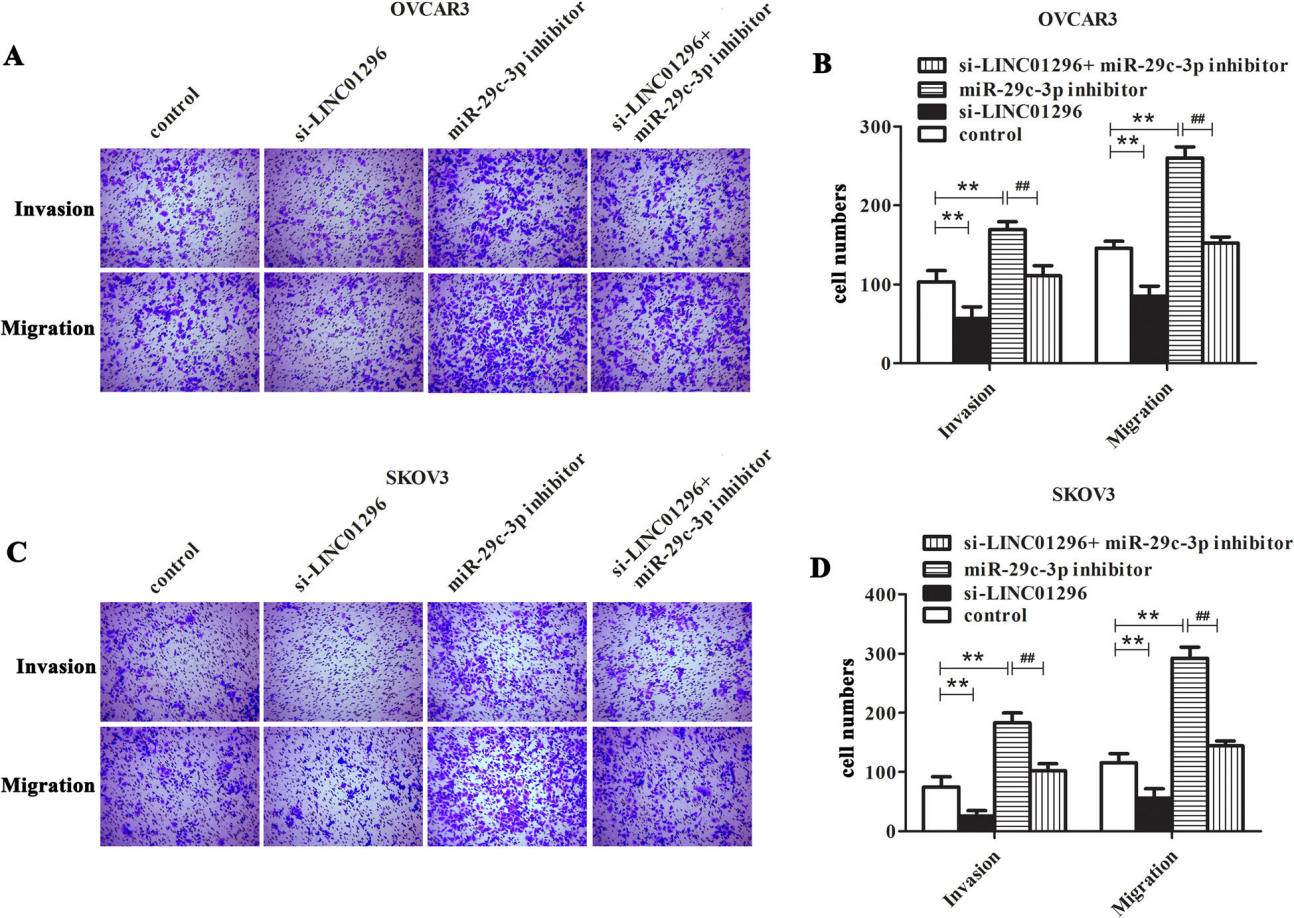
The influence of LINC01296/ miR-29c-3p axis on the invasion and migration of OC cells. We treated the SKOV3 and OVCAR3 cells with miR-29c-3p inhibitor, si-LINC01296, or miR-29c-3p inhibitor plus si-LINC01296. Transwell assay (**a**-**d**) was carried out to test the invasion and migration of OC cells in different conditions. **P < 0.01 vs. control group, ^##^*P* < 0.01 vs. miR-29c-3p inhibitor group

So as to assess the effects of LINC01296 down-regulation and miR-29c-3p inhibition on OC cells, the OVCAR3 and SKOV3 cells were transfected with si-LINC01296, miR-29c-3p inhibitor or si-LINC01296 plus miR-29c-3p inhibitor. Cells treated with transfection reagent were used as control. Previously, we demonstrated effective knockdown of LINC01296 [6]. Then CCK8, colony formation and Transwell experiments were performed in OVCAR3 and SKOV3 cells to detect the cell proliferation, invasion and metastasis. The results of CCK8 and colony formation assays were revealed that inhibition of miR-29c-3p can obviously increase the proliferation of OVCAR3 and SKOV3 cells compared with the control group (Fig. 3a-f, *P* < 0.01). On the contrary, down-regulation of LINC01296 can significantly reduce the cell proliferation compared with the control group (*P* < 0.01). In addition, we can see from the Fig. 3a-f that after OVCAR3 and SKOV3 cells co-transfected with miR-29c-3p inhibitor and si-LINC01296, cell proliferation showed a significant decrease compared with miR-29c-3p inhibitor group (*P* < 0.01). The Transwell experiments displayed the similar results that when treating OVCAR3 and SKOV3 cells with si-LINC01296 in combination with miR-29c-3p inhibitor, the inhibitory effects can be observed on cell invasion and migration than that being treated with miR-29c-3p inhibitor alone (Fig. 4a-d, *P* < 0.01). Taken together, these results suggested that down-regulation of LINC01296 can reverse the effect of miR-29c-3p suppression on OC cells and LINC01296 functions as a cancer-promoting gene in OC via regulating miR-29c-3p expression.

### Knockdown of LINC01296 reverses the miR-29c-3p-mediated inhibition on the epithelial mesenchymal transformation (EMT) in OC cells

**Fig. 5:**
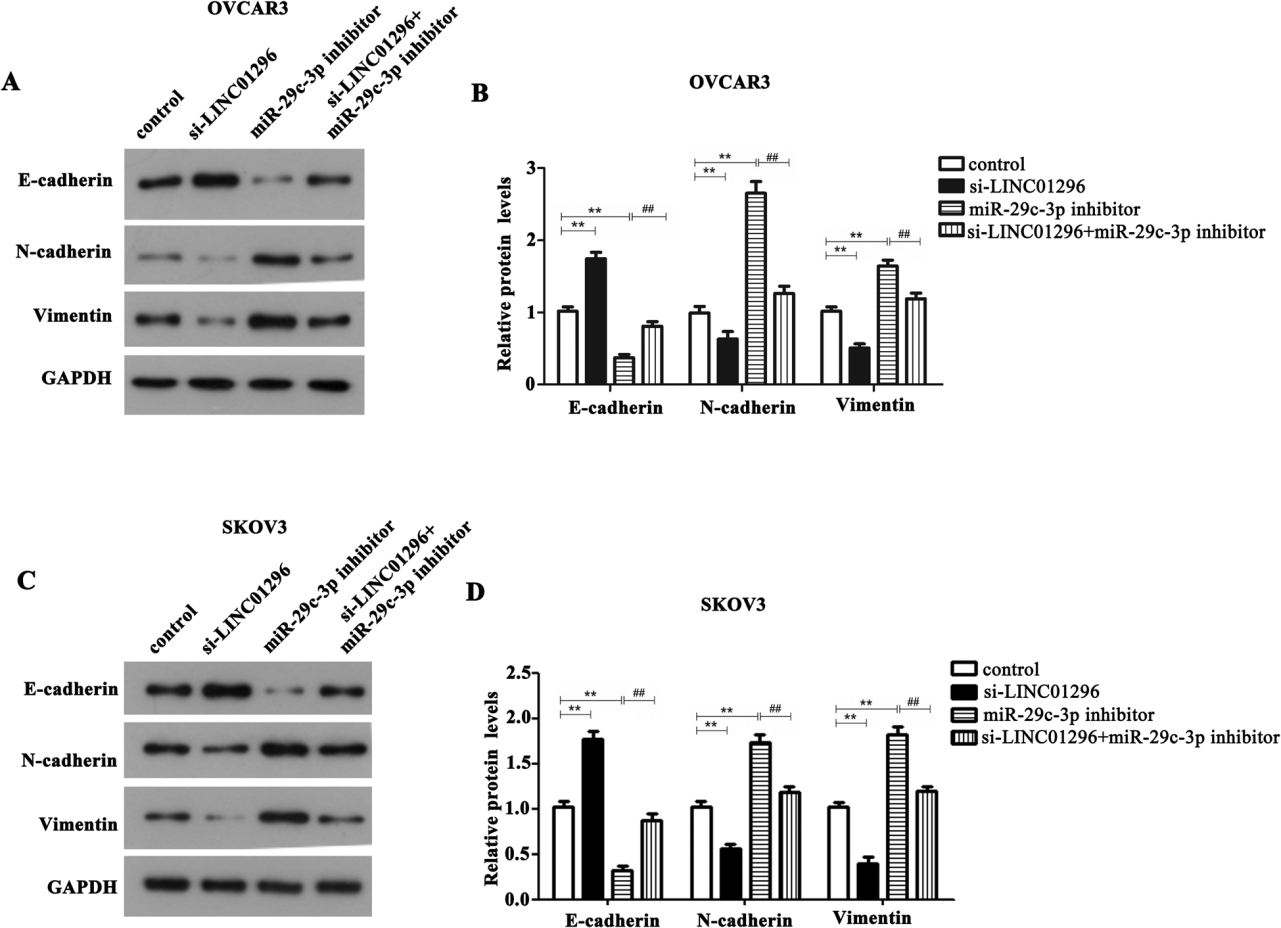
The influence of LINC01296/ miR-29c-3p axis on EMT pathway of OC cells. (**a**-**d**) The result of western blot analysis demonstrated that down-regulation of LINC01296 inhibited the EMT of OC cells, while inhibition of miR-29c-3p promoted the EMT of OC cells. When SKOV3 and OVCAR3 cells were treated with miR-29c-3p inhibitor plus si-LINC01296, the EMT was markedly suppressed compared with the miR-29c-3p inhibitor group. **P < 0.01 vs. control group, ^##^*P* < 0.01 vs. miR-29c-3p inhibitor group

EMT is essential for tumorigenesis and metastasis [16]. So we detected the changes in EMT-related proteins to in-depth explore the underlying mechanism of LINC01296/miR-29c-3p axis involved OC progression. The results obtained from the western blot analysis were shown in Fig. 5a-d. Loss of E-cad is a marker of EMT [17], and inhibition of miR-29c-3p can significantly decrease its expression in OVCAR3 and SKOV3 cells, while induce the expression of N-cadherin and Vimentin. In the meantime, knockdown of LINC01296 showed an opposite effect. Furthermore, after OVCAR3 and SKOV3 cells co-transfected with si-LINC01296 and miR-29c-3p inhibitor, we revealed that down-regulation of LINC01296 reverses the influence of miR-29c-3p inhibition on the procession of EMT in OC cells. Based on the above results we discovered that LINC01296 /miR-29c-3p axis mediates the mechanical regulation of EMT in OC.

## Discussion

In the past decade, non-coding RNA (ncRNAs) has been extensively studied in many biological processes and human diseases including cancer [9]. The expression of LncRNAs with carcinogenic characteristics is disordered in various of tumors [18], which promotes the metastasis and growth of cancer cells, thus playing a carcinogenic role [19, 20]. Our previous experiments have found that LINC01296, as an oncogene, was highly expressed in OC tissues and cell lines.

Similarly, miRNAs, as major regulators of gene expression in eukaryotes, have also been shown to function in the pathogenesis of various cancer [21]. The miR-29 family is an important member of miRNAs, which has the dual characteristics of carcinogenic and tumor suppressor, and can involve in various pathological procedures such as tumor growth and apoptosis [14]. For instance, in renal cell carcinoma, miR-29 can inhibit the migration and invasion of RCC cells in vitro by negatively regulating the expression of LOXL2 [22]. Similarly, miR-29c also showed anti metastasis effect in pancreatic cancer, which reduced the invasion and metastasis of pancreatic cancer cells by targeting MMP2 in vitro [23]. Moreover, miR-29 may also inhibit the expression of some genes involved in EMT and metastasis [24]. MiR-29c pertains to the miR-29 family and has been identified as markedly down-regulated microRNAs in cancer [14, 15]. It plays a tumor suppressive role in multiple cancers, such as colorectal cancer [25], lung cancer [26], breast cancer [27], gastric cancer [28], and pancreatic cancer [29]*.* Our experimental data further confirms this conclusion. In contrast to the expression of LINC01296 in OC, we found that miR-29c-3p was lowly expressed in OC tissues and cell lines, and down-regulation of miR-29c-3p was interrelated to poor outcomes.

Furthermore, it is noteworthy that more and more experimental evidence showed that there is a link between microRNAs and lncRNA, and some lncRNAs can target miRNAs to reduce their stability [8, 9]. In this paper, our data indicated that LINC01296 directly targets miR-29c-3p and negatively regulates the expression of miR-29c-3p. To explore the role of miR-29c-3p in OC, miR-29c-3p inhibitor was synthesized. The results illustrated that inhibition of miR-29c-3p enhanced the proliferation, migration and invasion of OC cells, which was consistent with the knockdown of LINC01296. In addition, we verified through the rescue experiment that knockdown of LINC01296 can reverse the promotion effect of miR-29c-3p downregulation on cell proliferation, invasion, migration. Together these results implied that LINC01296 functions as a cancer-promoting gene in OC via regulating miR-29c-3p expression.

Epithelial-mesenchymal transition (EMT) is the most important cellular biological process in the process of natural transdifferentiation, which plays a major role in the normal physiological development and wound healing of human body [14, 30]. Simultaneously, it also manifests itself in the process of tumor metastasis [14]. Heerboth S et al. considered that tumor metastasis occurs when cancer cells undergo EMT [31], while Xin Ye et al. believed that activation of EMT programs in tumor cells can promote the development of almost all malignant tumor-related features [32]. Increasing evidence suggests that inhibiting EMT pathway can inhibit metastasis, recurrence or drug resistance of tumors [33]. In our study, knockdown of LINC01296 inhibited the EMT of cells, while inhibition of miR-29c-3p promoted the EMT of cells. In addition, downregulation of LINC01296 and downregulation of miR-29c-3p showed a opposite effect on EMT, hypothesizing that imbalance of EMT-related genes may be involved in the LINC01296/miR-29c-3p axis-related migration and invasion of OC cells.

In sum up, our study demonstrated that the miR-29c-3p was down-regulated in OC and the lowly expression of miR-29c-3p was associated with poor outcomes. Moreover, LINC01296, as an oncogene in OC, regulates the biological function of OC cells by targeting miR-29c-3p, which also mediates the phenotype of EMT in OC cells. These findings supplied novel biomarkers and possibilities for OC therapy, but the potential application in clinical treatment still needs further exploration.

## Data Availability

The datasets during and/or analysed during the current study available from the corresponding author on reasonable request.
